# Preventing the preventable through effective surveillance: the case of diphtheria in a rural district of Maharashtra, India

**DOI:** 10.1186/1471-2458-13-317

**Published:** 2013-04-08

**Authors:** Revati K Phalkey, Rajesh V Bhosale, Abhijeet P Joshi, Sushil S Wakchoure, Muralidhar P Tambe, Pradip Awate, Michael Marx

**Affiliations:** 1 Institute of Public Health (Former Department of Tropical Hygiene and Public Health) Im Neuenheimer Feld 324, University of Heidelberg, Heidelberg, D-69120, Germany; 2 District Surveillance Office, Integrated Disease Surveillance Project, Ministry of Health and Family Welfare, Dhule, Maharashtra, India; 3 Joshi Hospital, Pune, Maharashtra, India; 4 Department of Preventive and Social Medicine, SBH Govt. Medical College, Dhule, Maharashtra, India; 5 Integrated Disease Surveillance Project, Ministry of Health and Family Welfare, Pune, Maharashtra, India

**Keywords:** Dhule district, Diphtheria, India, Maharashtra, Outbreak investigation, Response

## Abstract

**Background:**

Epidemic diphtheria is still poorly understood and continues to challenge both developing and developed countries. In the backdrop of poor immunization coverage, non-existent adult boosters, weak case based surveillance and persistence of multiple foci, there is a heightened risk of re-emergence of the disease in epidemic forms in India. Investigating each outbreak to understand the epidemiology of the disease and its current status in the country is therefore necessary. Dhule a predominantly tribal and rural district in Northern Maharashtra has consistently recorded low vaccination coverages alongside sporaidic cases of diphtheria over the last years.

**Methods:**

This study reports the findings of an onsite survey conducted to assess a recent outbreak of diphtheria in Dhule district and the response mounted to it. Secondary data regarding outbreak detection and response were obtained from the district surveillance office. Clinical data were extracted from hospital records of eleven lab confirmed cases including one death case. Frequency distributions were calculated for each identified clinical and non- clinical variable using Microsoft™ Excel® 2010.

**Results:**

Our findings suggest a shift in the median age of disease to adolescents (10-15 years) without gender differences. Two cases (18%) reported disease despite immunization. Clinical symptoms included cough (82%), fever (73%), and throat congestion (64%). About 64% and 36% of the 11 confirmed cases presented with a well defined pseudomembrane and a tonsillar patch respectively. Drug resistance was observed in all three culture positive cases. One death occurred despite the administration of Anti-Diphtheric Serum in a partially immunized case (CFR 9%). Genotyping and toxigenicity of strain was not possible due to specimen contamination during transport as testing facilities were unavailable in the district.

**Conclusions:**

The outbreak raises several concerns regarding the epidemiology of diphtheria in Dhule. The reason for shift in the median age despite consistently poor immunization coverage (below 50%) remains unclear. Concomitant efforts should now focus on improving and monitoring primary immunization and booster coverages across all age groups. Gradually introducing adult immunization at ten year intervals may become necessary to prevent future vulnerabilities. Laboratory networks for genotyping and toxigenicity testing are urgently mandated at district level given the endemicity of the disease in the surrounding region and its recent introduction in remote Dhule. Contingency funds with pre- agreements to obtain ADS and DT/Td vaccines at short notice and developing standard case management protocols at district level are necessary. Monitoring the disease, emerging strains and mutations, alongside drug resistance through robust and effective surveillance is a pragmatic way forward.

## Background

Diphtheria is endemic in most developing countries including India. It is transmitted through aerosols and is communicable for two to six weeks in the absence of antibiotic treatment [1-4]. The incubation period is two to five days, however symptoms may develop up to ten days after exposure. Cyclic epidemics of diphtheria have been documented since the 16th century in both developed and developing countries. The most striking resurgence was witnessed in the nineties in Eastern Europe [5]. The main reasons for the re-emergence of diphtheria in the post vaccine era are waning of immunity with age and improved immunization rates in children which reduces circulating wild organisms and hence the opportunities for natural boosting through subclinical infections [6].

As a result, a shift in the median age of incidence to adolescents and adults has been reported from several countries [6-8]. Furthermore, cases and deaths have been documented in previously immunized individuals [9]. *Epidemic* diphtheria is still poorly understood and immunization to date remains mainstay for prevention. There is no standard vaccination schedule for diphtheria, although all schedules deliver a primary series consisting of three doses starting at a minimum age of six weeks with a minimum interval of four weeks between the doses. Choice for the timing and number of boosters depends mainly on other diseases in the combination vaccines used in the respective Expanded Program for Immunization (EPI) programs and on the sero-epidemiology and endemicity of the disease in each country [10].

India accounted for over 71% of the 4880 diphtheria cases in the world in 2011 [11]. Although this is partly accounted for by disproportionate size of the Indian birth cohort and influenced by variable reporting efficiency of diphtheria cases between countries, the disease remains largely neglected and widely prevalent with periodic intermittent outbreaks in over 12 states of the country [1-4,9,12-24]. Given the persistence of multiple foci in all regions of the country there is indeed a heightened risk of re-emergence of the disease in epidemic forms. An improved understanding of the nature of each outbreak is therefore mandated to generate a pool of evidence regarding the current disease status in India. This paper aims to further existing knowledge about diphtheria in the Western state of Maharashtra.

We report an assessment of the August 2011 outbreak in Pashte village in rural tribal Dhule district, of Northern Maharashtra and the response mounted to it. Cases have been earlier reported from the region especially Malegaon block of neighbouring Nashik district [9]. However, this is the first time a geographically localized outbreak of significant size is reported from Dhule district. The main objective the study was to describe the nature of the disease itself and the outbreak in the region, identify the probable causes for persistence of the disease in the area and to highlight some strategies to prevent its recurrence. The paper aims to highlight the importance of the Integrated Disease Surveillance System/ Project (IDSP) in monitoring disease epidemiology and in detecting and responding to outbreaks in a structured and timely manner.

## Methods

### Study area

Pashte village lies in Sindhkhede block of Dhule district and covers a population of 3359. The Primary Health Care Center (PHC) Betawad serves a population of 34,492 including Pashte and is a reporting unit within the IDSP. It is manned by two Medical Officers, 11 paramedical staff at the headquarters and five satellite subcenters. The subcenter in Pashte is manned by one Auxiliary Nurse and Midwife. Dhule is one of the 34 districts in Maharashtra which implements the Integrated Disease Surveillance Project (IDSP) since 2005. The system uses syndromic, presumptive and lab confirmation approaches to collect data on cases of 21 priority diseases and syndromes through S, P and L reporting formats respectively from identified public and private reporting units on a weekly basis from both urban and rural areas including diphtheria [25]. The main objective of the system is outbreak detection and response. Every district has a Rapid Response Team which is activated as soon as an action threshold for a disease is reached which is a single confirmed case for diphtheria.

### GIS mapping

**Figure 1:**
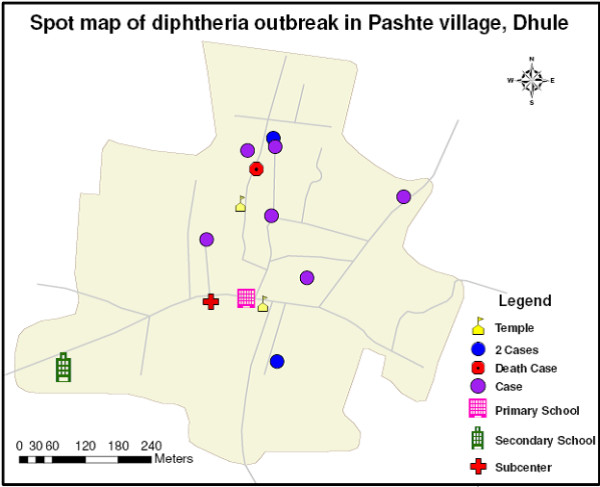
Spot map.

The location of households of the cases; the primary school; and the sub-center were identified and GPS coordinates were collected using a handheld e-Trex GPS machine after calibration. Location was collected in degree decimal (hddd.ddddd). The North reference was kept true. A point layer was generated using the GPS coordinates and was validated by overlaying the points on Google Earth which were then overlaid on the Pashte boundary map using ArcGIS version 9.3 to generate maps (Figure 1).

### Case definitions

We classified cases as per the WHO recommendations [26]. A probable case was defined as a person having laryngitis or pharyngitis or tonsillitis, and an adherent membrane / patch of the tonsils, pharynx and/or nose. Confirmed case was defined as a probable case with lab confirmation (isolation of *corynebacterium diphtheriae* from a clinical specimen) as smear and/ or culture positive.

### Data processing and analysis

Secondary data regarding outbreak detection, investigation and response was collected from the Integrated Disease Surveillance (IDSP) office in Dhule district. Clinical data on identified treatment and outcome variables were extracted from hospital charts and discharge case reports of 11 lab confirmed cases by a consultant physician in general medicine (nine cases including the death case treated at the medical teaching (civil) hospital in Dhule and two cases treated at the Kasturba Hospital for Infectious Diseases, Mumbai). The data were entered, cleaned and analyzed in Microsoft™ Excel® 2010. Frequency distributions showing number and percentages were generated for each identified clinical and non-clinical variables

### Ethics approval

The study was a part of the doctoral dissertation of the lead author and was passed through the Ethical Committee of the Medical Faculty, University of Heidelberg, Germany (Approval Number: S381/2010) and by the University of Delhi, India (Approval Number: Anth/2009/585). Additionally relevant permissions and consents were obtained from the District Health Officer (DHO), the Gram Panchayat (village administration) members and the village leaders. Informed verbal consent was obtained from all individuals from whom swabs were collected.

## Results

### Timeline of outbreak

A six year male child presented at medical teaching hospital, Dhule with one day history of fever, cough, sore throat, difficulty in swallowing and breathing, congestion in throat and swelling in neck (*bull’s neck*) along with a well formed pseudo-membrane with pre-admission consultation with an Ear Nose Throat (ENT) consultant on July 28, 2011. Diagnosis was confirmed by clinical examination and a positive smear and culture. Anti Diphtheria Serum (ADS) was administered single dose 80,000 IU intravenously over six hours immediately along with 6.6 LIU crystalline penicillin six hourly. The condition worsened despite ADS and the patient passed away on August 2, 2011- five days post admission. Death was informed to District Health Officer (DHO) and District Surveillance Officer (DSO) under the IDSP which immediately triggered actions at district, block and village levels. The district Rapid Response Team (RRT) was activated and house to house surveys were conducted for two weeks and over 560 school children (0–6 years) were screened. No clinically probable cases were identified. Mass prophylaxis with Azithromycin (10 mg/kg) dose adjusted to age and weight was administered to all fever cases.

A second wave started six weeks later with the diagnosis of a six year old male (cousin of the index death case) on September 19, 2011, reported over telephone to IDSP, triggering a second phase of control measures. Surveys were conducted in 633 households and the primary and secondary schools. A short questionnaire was designed and all concerned health staff from the PHC and subcenters were trained in screening and data collection for the house to house survey and a medical camp. All households in the village were covered by three teams and all 2100 individuals in the village at time of outbreak were screened. Clinically confirmed cases and their contacts were continuously tracked. Additionally other health conditions were attended to during the survey and attempts were made to obtain verbal vaccination history or cards during the visits. Respiratory etiquette, hand hygiene was advocated. Ninety one cases with history of sore throat (29.6%); fever (66.0%); running nose (63.7%); or pain on swallowing (44%) were identified during the house to house visits by paramedical staff and referred to the subcenter for further clinical examination.

An onsite medical camp was conducted by the district IDSP Rapid Response Team (RRT) that included a paediatrician, microbiologist, two lab technicians, ENT consultant, epidemiologist and a public health specialist from the medical teaching hospital. On arrival 85 throat swabs were obtained for bacterial culture as microbiologist and lab technicians were available on-site and in order to avoid individual travel to the district hospital which is 45kms away. The team also took a broad all inclusive approach to treatment by administering Azithromycin (10 mg/kg) dose adjusted to age, and weight for seven days. Further, detailed examination by paediatricians and internists identified ten probable cases that met clinical suspicion with either a tonsillar patch or a well-defined membrane and were isolated for hospital admission.

### Bacteriology

Samples were collected and transported in sterile test tubes by qualified technicians and microbiologists from the Government medical teaching hospital laboratory. Staining techniques included Gram’s staining followed by Albert’s staining. Culture techniques included inoculation using streaking on blood agar medium and blood potassium tellurite agar was used as the selective medium. Antimicrobial susceptibility was tested using Kirby-Bauer (disc diffusion method) with American Type Culture Collection (ATCC) strains of gram positive and gram negative organisms as controls. All ten probable cases were lab confirmed. The results were positive for *C. Diphtheriae* in nine smears and two cultures. One case was smear negative but culture positive. Including the death case (which was both smear and culture positive) we report results of 11 lab confirmed cases in our study. Samples were sent for bio-typing and toxigenicity testing to the referral lab under IDSP in Pune, but were contaminated and hence unfit for processing. All three culture positive samples were found resistant to penicillin, ampicillin, cefuroxime, and ciprofloxacin.

### Age and gender distribution

Six of 11 (54.5%) lab confirmed cases (including the death case) were males and five were females (45.5%). Attack rates increased with age and were highest for the age group 10–15 years. Gender distribution remained similar across age groups (Table 1). The median age of the ten smear positive cases was 11 years (range 5–15 years) and that of the three culture positive cases was 12 years (range 6–13 years).

**Table 1:** Age and sex wise attack rates of confirmed cases

**Age group**	**Males (n)**	**Confirmed cases**	**Attack Rate (%)**	**Females (n)**	**Confirmed cases**	**Attack Rate (%)**	**Total population (N)**	**Total Confirmed cases**	**Attack rate (%)**
**0 – 5**	179	1	0.6	173	1	0.6	352	2	0.6
**6 – 10**	111	1	0.9	106	1	0.9	217	2	0.9
**10 – 15**	141	4	2.8	129	3	2.3	270	7	2.6
**15 – 45**	796	0	0	723	0	0	1519	0	0
**45 and above**	514	0	0	487	0	0	1001	0	0
**Total**	1741	6	0.3	1618	5	0.3	3359	11	0.3

### Vaccination status

Attempts made to obtain vaccination coverage estimates for the village through registers at the PHC, vaccination diaries of subcenter and PHC staff and the National Rural Health Mission (NRHM) Reproductive and Child Health II (RCH II) databases were in vain due to incomplete documentation and monitoring of targets instead of actual number of children vaccinated. Secondly, parental recall and vaccination cards were sought during two separate visits but were also unsuccessful. Nonetheless, primary immunization status was confirmed in two of the ten (20%) survivors- one verbally and one supported by a vaccination card). Reports of the death case showed that DPT1 and DTP2 were administered in private facility and records for DPT3 and 1st booster were recovered from the PHC confirming that the child was at least partially immunized.

### Pre-admission consultation and duration of hospitalization

Over 45.4% cases admitted having consulted private practitioner for a second opinion (mostly with ENT consultants and one with an ayurvedic practitioner) before being admitted to the medical teaching hospital. Duration for hospitalization ranged from 1–15 days and maximum (54.5%) cases were hospitalized for six days.

### Clinical symptoms and treatment outcomes

**Figure 2:**
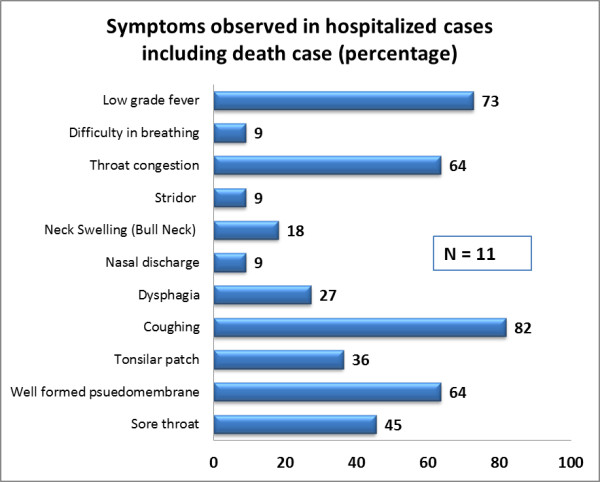
Frequency of reported clinical symptoms.

Cough (82%), low grade fever (73%), and throat congestion (64%) were the most frequently reported clinical symptoms (Figure 2). The death case presented with high grade fever. Over 90% of the cases were faucial or tonsillar type and 64% and 36% cases presented with a well formed pseudomembrane and a tonsillar patch respectively. Two cases presented with classical neck swelling (*bull’s neck)*. Only one case progressed to respiratory complications and died (CFR 9%). Neurological or cardiac complications did not occur in any of the cases.

### Treatment

ADS was administered to all patients immediately on admission. The ADS dose used for treatment ranged from 40,000 IU to 100,000 IU according to severity of disease and was administered intravenously in a single dose over four to six hours. Three children developed mild reactions that included vomiting and puffiness of eyes. ADS was stopped in one child after four test doses. Mono-therapy with crystalline penicillin (12,500-25,000 IU/kg) and in cases of resistance or intolerance Azithromycin (10 mg/kg) was included in the regimen. The duration of antibiotic therapy was ten days in seven cases and seven days in two surviving cases. One case (documented history of primary immunization) was treated with IV antibiotics for 14 days. Only two cases were vaccinated before discharge due to unavailability of the DT vaccine. Chemoprophylaxis with Azithromycin (10 mg/kg body weight) was administered to all contact cases for three to ten days.

## Discussion

The outbreak provides a realistic description of how the district authorities detected and reacted to the event. Although the first response mounted was fairly reasonable the incident raises several concerns regarding the epidemiology of diphtheria itself in the district as well as the preparedness of the primary healthcare system. A remarkable feature of this outbreak was recognition of the risk of re-emergence of diphtheria by the district authorities and moreover their proactive initiative to investigate the problem and to plug gaps. Part of the credit goes to the Integrated Disease Surveillance System which currently being the only significant large scale surveillance effort in the state is paramount in raising awareness regarding surveillance of emerging and re-emerging diseases. Further, not only does IDSP provide training and equipment support to the staff, but also ensures contingency funds for outbreak investigations, making it rather indispensable in disease monitoring at local levels.

### The age paranoia

The attack rates observed in our study increased with age and were highest in the 10–15 years age group. This re-confirms findings from earlier studies in the region (57.1% in 5–14 years age) [9]. Ray et al. reported a shift in the median age of diphtheria in Calcutta, in as early as 1998 and attributed the shift to good primary immunization [3]. Age shift was reported from Karnataka in 1992 [13] and Andhra Pradesh between 2003–2006 [12]. A recent outbreak in Assam reported over 70% of the cases between 15- 45 years of age confirming the upward trend for disease incidence in all regions of the country [20]. Although the size of the outbreak does not support any statistical claims for the issue, it does serve as an early warning for the region and should not be ignored. The reasons for the observed age shift in Dhule are a challenge to be investigated.

Age shift, is considered an indicator of good under five immunization [27]. This is however not the case in Dhule given that the vaccination coverage has been consistently low over years. As per District Level Household and Facility Survey (DLHS 2007/2008) coverage of DPT3 was 45.2% and the BCG- DPT3 dropout rate stood at 14.6% in the district [28]. It is also prudent to recognize that the coverage thresholds required for herd protection to eliminate disease are not necessarily the same as those that will result in a shift in the age, and could in fact be lower.

Furthermore, age shift has also been documented before mass immunizations were instituted and factors such as migration, nutritional status, displacement, socio-economic conditions, coexistence of large groups of susceptible children and adults, and deterioration of health infrastructure played a role and in fact may offer partial explanation to our observations given the tribal and predominantly rural nature of the area [7,29,30].

Another plausible explanation is the improved primary immunization rates in the last five years post introduction of the National Rural Health Mission (NRHM). This may actually have led to susceptible adolescents outnumbering the number of susceptible children [1]. Joint WHO-UNICEF and other national and state surveys peg the coverage between 60–80% between 2005–2009 respectively (Table 2). However, a minimum coverage of 90% in children under five and 75% in adults is necessary to confer herd immunity and current coverage rates in the district remain rather inadequate [31]. Additionally, why the age shift is limited to adolescents despite susceptible adults remain unclear.

**Table 2:** DPT3 coverage estimates for India and Maharashtra state (in%)

	**2004**	**2005**	**2006**	**2007**	**2008**	**2009**	**2010**	**2011**
Official Government Estimate (India)	87	90	94	98	84	97	90	85
WHO-UNICEF Joint Estimate (India)	64	67	66	71	72	72	72	72
DLHS 2 and 3 (Maharashtra)	72	-	-	-	79	-	-	-
DLHS 2 and 3 (Dhule district)	88	-	-	-	45	-	-	-

(Source [11,32-34])

Despite the awareness of the issue in the region, accurate data on immunization coverage were unavailable. The routine immunization monitoring system calculates rates as per targets (number of children vaccinated) provided by the state and reports over vaccination by 10- 12% [35]. Standardized monitoring mechanisms with routine data quality and accuracy checks for appropriate denominators may help improve understanding of actual coverage.

Other factors that contribute to the low immunization coverage include lack of awareness, misconceptions, avoiding immunizations for trivial reasons, migration, declining enthusiasm to routine immunization and unilateral focus on polio campaigns [2]. Health worker attitude and skills, vaccine condition, and cold chain influence vaccine success and regular audits must be made part of monitoring and evaluation activities. Supportive supervision of immunization practices by external consultants and medical colleges has demonstrated significant improvements in coverage and may be of added value in the district [36].

### The booster dilemma

The Universal Immunization Program (UIP) in India offers three doses of DwPT at six, ten and fourteen weeks and two booster doses at 16–24 months and five years of age [11]. Over 94-100% children develop protective levels of antibodies following primary immunization but in the absence of boosters the titres drop and over 20–65% of adults may become susceptible [5,30,37,38]. Waning of antibodies with age has been documented through serological surveys in both developing and developed countries including India [6,39]. Immunization schedule especially the spacing of boosters play an important role in maintaining lifelong immunity [8,40]. DT boosters throughout adult life at ten year intervals and minimum two booster doses over 40 years of age are recommended [41-44].

The need to revise the UIP is urgent and has been discussed before [16]. Adult boosters at ten year intervals are currently non-existent in the country [45] and could be considered *vis-a-vis* the resource availability and urgency. DT booster at school entry and leaving should be considered to cover adolescents. Serological studies for antibody titres in adults and adolescents can assist in identifying the timing and number of booster doses for the district [10]. Secondly, data on booster 1 and 2 coverage is not collected routinely [46]; or reported to the WHO. This calls for moving beyond primary immunization and the inclusion of DPT 1 and 2 booster coverage as an indicator of health system performance under NRHM.

### Immunized or not- does it really matter?

We report invasive disease in a child with documented primary immunization and death in a partially immunized child. Disease despite primary immunization has been reported earlier from Rajkot, Gujarat [2] and from Malegaon and Dhule district [9]. The death of the child despite ADS was due to advanced clinical symptoms at admission and the fact that the antitoxin only neutralizes circulating toxin that is not still bound to the tissue [5]. The disease was probably also more severe due to incomplete immunization [23,24]. Case fatality increases with late presentation, delay in diagnosis and non-availability of ADS [35,47]. None of which were true in our case. Death occurred due to upper airway obstruction which is the commonest complication in children due to their small airways [48]. Issues such as vaccine quality and / or vaccine failure should be investigated given that cases with previous DPT doses have reported the disease in the district [16].

Vaccination history was unavailable for a majority of the cases in our study like in most others [49]. Recording and updating immunization status on birth or school leaving certificates or the recently issued *Aadhaar* cards (Unique Identification Authority of India) that carry weight throughout adult life may help resolve the issue

### Biotype, toxigenicity and sensitivity

Genotyping diphtheria strains not only allows differentiation of endemic, epidemic, and imported strains but also helps monitor its adaptability, virulence, and pathogenicity, thus ensuring adequacy of protection through existing vaccines [50]. Few published studies in India report the biotype or toxigenicity [17,21]. It was not possible to conduct toxigenicity or strain identification on the samples in our study due to contamination which is a serious limitation. Elek facilities were unavailable at district headquarters and even in the identified referral lab under IDSP. Laboratory strengthening for endemic diseases is especially vaccine preventable diseases like diphtheria currently indispensable.

Frequent isolation of non-toxigenic strains in immunized individuals may offer an explanation to disease in previously immunized children in our study [51]. In India, two separate events of non-toxigenic strain isolation have been reported in Pondicherry [17,22]. Non-toxigenic strains associated with invasive disease have also been found resistant to cefotaxime and ciprofloxacin [52]. However, a study from North India reports 83.3% of the 54 isolates- 90% of which were toxigenic and biotype *var intermedius* were resistant to trimethoprim /sulpha methaxazol, 18.5% to ampicillin and one to ciprofloxacin [21]. The correlation between toxigenicity, biotype and drug resistance is therefore unclear.

An earlier study from Dhule district reported biotype gravis as the most common strain (1991–2007) and all isolates were sensitive to penicillin and erythromycin [9]. However, all three culture positive samples in our study including the death case were resistant to penicillin, ampicillin, cefuroxime, and ciprofloxacin which is a significant concern particularly in a predominantly rural area where the disease has probably been long endemic. In the absence of strain and toxigenicity data it is premature to comment on the resistance to drugs found in our study. Nonetheless, the seriousness of the issue cannot be undermined and emerging resistance, if any, should be closely monitored and clinicians regularly updated.

### Clinical management and patient outcomes

We report ten smear and three culture positives and one case of smear negative and culture positive probably because of poor sample collection techniques [18]. Over 50% of the culture negative cases had been administered antibiotics before sample collection which calls for refresher training and developing job aids for sample collection and processing particularly at the field level.

Despite the shift in median age, clinical features remain largely similar to those observed in the pre-vaccine era [6,18] unlike cases in the Eastern Europe epidemic where only 32.5% of the cases presented with a classical membrane [8,53]. Seven cases (64%) in our study presented with a well formed membrane and four (36%) with a tonsillar patch. Physicians, both public and private should be sensitized to maintain a high index of suspicion for diphtheria even in the absence of classical symptoms and regular reminders through Continuing Medical Education (CME) sessions should be organized as a reminder of disease endemicity.

All ten cases in our study recovered completely and were asymptomatic on discharge. In 2005, seven deaths (CFR 10%) were reported from Dhule and Malegaon districts due to the non-availability of ADS [9,54]. A CFR of nine per cent in our study despite availability of ADS is unacceptable although we do report difficulty in obtaining ADS at short notice. Vaccination at discharge was not possible due to unavailability of the DT vaccine. Maintaining contingency funds for local purchases and pre-agreements with local vendors to obtain ADS and DT vaccines at short notice during outbreaks should be considered in district preparedness plans.

The duration of antibiotic therapy ranged from seven to ten days in cases and three to five days in contacts in our study which should be revised to the current guidelines of 14 days treatment to both. Repeat swab for microbial confirmation at discharge was not done. More importantly, standard case management protocols need to be developed for diphtheria and regularly updated based on drug sensitivity testing for the district.

### Surveillance is the key

Absence of mandatory case based reporting, weak laboratory networks, tendency of peripheral staff to under/non report cases due to fear of action from superiors and weak data analysis at the district level failed to provide a real picture of the disease burden in Dhule district in the past. Despite regular sporadic incidence of disease, institutional learning has been poor.

The outbreak successfully demonstrated the strengths of IDSP in early detection and reporting of emerging diseases. Given that the outbreak was localized, large and geographically within the district it was successful in drawing attention. Effective control and prompt response helped cap the fatality rate. Routine surveillance has now improved through regular supervision by the district surveillance unit and although under- reporting still exists, review of data and its quality is improving steadily. The outbreak validates the fact that surveillance is indispensable in furthering our understanding of changes in both the organism and its victims and this should be strongly advocated at all levels.

#### Limitations of the study

We report data for one particular outbreak especially when diphtheria is endemic in the district with sporadic cases reported for several years. Immunization history and vaccination cards were unavailable in majority of cases despite attempts made to recover them. Parental recall was also poor. Strain identification and toxigenicity testing was not possible due to sample contamination. Although the use of retrospective secondary data and especially from an outbreak of this size does not allow generalizations to other areas or populations, there are some issues that could be common and help discretionary collateral learning. Better documentation of the onset, progress and control of outbreaks in the district is necessary to allow institutional learning.

## Conclusion

The outbreak confirms the upward trend in median age of diphtheria incidence observed in other states of India. Reasons for the shift only to adolescents despite consistent poor immunization coverage and a sufficient pool of susceptible adults - remain unclear. Vaccination programs in the district need to be assessed for efficiency and stepped up to prevent future outbreaks. Concomitant efforts should focus on improving and monitoring primary immunization and booster coverages across all age groups. Gradual introduction of adolescent and adult immunization at ten year intervals may become necessary as the next step to a long term futuristic strategy. Laboratory networks for genotyping and toxigenicity testing at district headquarters requires strengthening. Contingency funds and pre- agreements with local vendors to obtain ADS and DT/Td vaccines at short notice during outbreaks and standard case management protocols will improve patient outcomes and arrest case fatality rates. Monitoring disease trends, clinical progression, emerging strains and mutations, alongside drug resistance through robust and effective surveillance is the pragmatic way forward.

## Competing interests

The authors declare that they have no competing interests.

## Authors’ contributions

RP designed the study, conducted the fieldwork, analysed data and drafted the manuscript. RB conducted fieldwork and performed the statistical analyses. AJ carried out the collection and analysis of the clinical data and contributed to drafting of the manuscript. SW, PA and MT supervised the field study, assisted with data access, analysis and provided inputs to the manuscript. MM designed the study, contributed to the manuscript supervised the whole process. All authors read and approved the final manuscript.

## Author’s information

RP has completed her Masters in International health from Humboldt University in Berlin and is currently a doctoral student at the Institute of Public Health, University of Heidelberg, Germany. RB has completed his Masters in Bio-Statistics from the University of Pune and is working as district epidemiologist at the District Surveillance Unit (DSU) in Dhule, Maharashtra, India. AJ is a MD with DNB in Internal Medicine and heads the intensive care unit at a hospital in Pune, India. SW is MD in Preventive and Social Medicine and works as the District Surveillance Officer at the DSU, Dhule, India. MT is the Professor and Head, Department of Preventive and Social Medicine at the SBH Govt. Medical College Dhule, Maharashtra, India. PA is a MD and works as the State Surveillance Officer in Maharashtra, India. MM is a MD with Diploma in Tropical Medicine and Public Health and works as an Assistant Professor at the Institute of Public Health, University of Heidelberg, Germany.

## Pre-publication history

The pre-publication history for this paper can be accessed here:

http://www.biomedcentral.com/1471-2458/13/317/prepub
